# Supplementary material for: ATG-3 limits Orsay virus infection in C. elegans through regulation of collagen pathways
Source: bioRxiv. 2025 Jan 13:2025.01.13.632696. Preprint. [Version 1] doi: 10.1101/2025.01.13.632696 (PMC11761658; doi:10.1101/2025.01.13.632696)
Supplement: 1 [file NIHPP2025.01.13.632696V1-supplement-1.pdf]

bioRxiv preprint doi: https://doi.org/10.1101/2025.01.13.632696; this version posted January 13, 2025. The copyright holder for this preprint (which was not certified by peer review) is the author/funder, who has granted bioRxiv a license to display the preprint in perpetuity. It is made available under aCC-BY 4.0 International license.

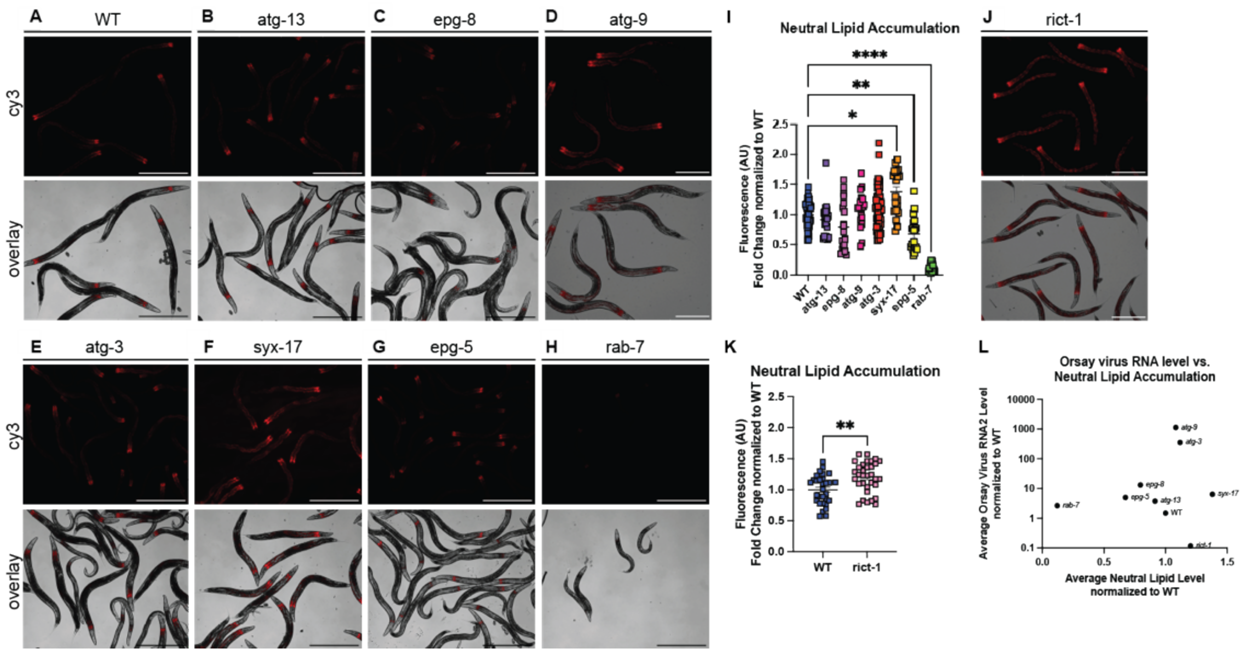

Supplemental Figure 1: Neutral lipid accumulation in a subset of autophagy mutants does not correlate with susceptibility to Orsay virus infection.

(A-H, J) Neutral lipid fluorescence microscopy was performed on day 3 adults that were synchronized as L1 embryos and plated on OP50 mixed with LipidTox dye. Scale bars represent 500 μM. **(I, K)** Quantification of neutral lipid fluorescence microscopy shown in panels A-H and J. Fluorescence (shown in arbitrary units (AU)) was normalized by setting the value of WT to 1. Each point represents one animal. Data are from 3 independent experiments performed with 3 replicates. Mean +/- standard error is shown. Statistically significant differences were determined by Kruskal-Wallis test with *post hoc* comparisons analyzed by Dunn's multiple comparison test (****, $p < 0.0001$; ***, $p < 0.001$; *, $p < 0.05$; ns = non-significant $p > 0.05$) when three or more samples were compared. When two samples were compared, statistical significance was determined by Mann-Whitney test (** = $p < 0.01$). **(L)** Average Orsay virus RNA levels plotted against average neutral lipid accumulation. Non-parametric Spearman correlation coefficient r=0.1167 with a p value of 0.7759.
